# Review of Therapeutic Potential of Coenzyme Q10 in Ophthalmology: Focus on Age-Related Macular Degeneration, Glaucoma, and Retinitis Pigmentosa

**DOI:** 10.3390/antiox15040506

**Published:** 2026-04-19

**Authors:** Michał Wiciński, Anna Fajkiel-Madajczyk, Zuzanna Kurant, Łukasz Rzepiński, Maciej Słupski

**Affiliations:** 1 Department of Pharmacology and Therapeutics, Faculty of Medicine, Collegium Medicum in Bydgoszcz, Nicolaus Copernicus University, M. Curie Skłodowskiej 9, 85-094 Bydgoszcz, Poland; michal.wicinski@cm.umk.pl (M.W.); zuzanna.kurant@cm.umk.pl (Z.K.); 2 Department of Neurology, 10th Military Research Hospital and Polyclinic, 85-681 Bydgoszcz, Poland; luk.rzepinski@gmail.com; 3 Department of Clinical Medicine, Faculty of Medicine, University of Science and Technology, 85-796 Bydgoszcz, Poland; 4 Department of Hepatobiliary and General Surgery, Faculty of Medicine, Collegium Medicum in Bydgoszcz, Nicolaus Copernicus University, M. Curie Skłodowskiej 9, 85-094 Bydgoszcz, Poland; maciej.slupski@cm.umk.pl

**Keywords:** coenzyme Q10, CoQ10, ophthalmology, AMD, RP, glaucoma

## Abstract

Coenzyme Q10 (CoQ10), a natural antioxidant produced by the human body, has strong anti-inflammatory properties, reduces oxidative stress, and improves mitochondrial function. It is also known for its strong neuroprotective effects. With age, endogenously produced CoQ10 levels decline, contributing to the development of chronic diseases, including eye disorders. Irreversible ocular diseases that result in blindness present a significant challenge in contemporary medicine, as no fully effective cure exists; current treatments primarily aim to decelerate disease progression, manage symptoms, and preserve residual vision. Our study reviews research on the use of CoQ10 in eye diseases like age-related macular degeneration (AMD), retinitis pigmentosa (RP), and glaucoma, which can cause permanent vision loss and are linked to oxidative stress and mitochondrial dysfunction. This article explores whether CoQ10 can be a safe and effective addition to treatment for these conditions. We also outline directions for future research and explain how CoQ10 functions in the studies discussed in this review.

## 1. Introduction

Due to advancements in medical science and improvements in the quality of life, the global population is aging. In 2024, people aged 65 and over accounted for 21.6% of the European Union (EU) population. Moreover, Eurostat forecasts a further increase in this percentage and notes a trend in which older people outnumber younger people in most member states [1]. Aging, as a complex and chronic process, also involves the breakdown of cell structures and their functions. This imbalance leads to the development of many chronic diseases. The World Health Organization (WHO) states that aging can be categorized as a disease in the International Classification of Diseases (ICD-11) [2]. Therefore, reducing the negative effects of aging and decreasing the likelihood of chronic diseases should be key areas of research for scientists and medical professionals.

It is well established that aging increases the risk of developing metabolic [3,4], cardiovascular [5,6], and neurodegenerative diseases [7,8]. Furthermore, as individuals age, the quality of vision often declines. For numerous ocular diseases, aside from genetic factors and other comorbidities, advancing age constitutes a significant risk factor. Eye tissues such as the lens, vitreous, and retina change with increasing oxidative stress and protein buildup in the lens as the body ages. This results in the development of cataracts and the decline in retinal pigment cell function associated with macular degeneration [9]. According to the WHO, at least 2.2 billion people suffer from vision impairment, which poses a global financial burden with an annual cost of $411 billion. The most affected are those over 50 years old [10]. Research shows that aging is highly associated with age-related macular degeneration (AMD), age-related cataracts, glaucoma, diabetic retinopathy (DR), and dry eye syndrome [9,11,12]. Moreover, research shows that many chronic diseases, including eye diseases, are closely linked to mitochondrial dysfunction. These conditions are associated with problems in mitochondrial energy production, increased oxidative stress, and elevated inflammation [13]. One such eye condition strongly associated with mitochondrial dysfunction is retinitis pigmentosa (RP) [14].

In recent years, there has been a notable increase in interest in healthy diets as methods for delaying aging and preventing chronic diseases. Currently, much focus is on natural antioxidants, which not only fight oxidative stress but also help slow the aging process [15]. One such natural substance with antioxidant properties is coenzyme Q10 (CoQ10), which is widely used in supplementation for many chronic diseases [16]. CoQ10 shows antioxidant properties by lowering reactive oxygen species (ROS) levels and preventing lipid peroxidation by stabilizing cell membranes [17]. It is produced in the human body through specialized pathways in the cytoplasm and mitochondria. The body’s production of CoQ10 declines with age, and deficiencies can also be linked to genetic defects and certain medications, such as statins. Additionally, lower levels of CoQ10 in tissues and organs are associated with specific chronic diseases [16]. Considering the complex pathogenesis of eye diseases related to mitochondrial dysfunction, oxidative stress, and aging [18,19], CoQ10 appears to be a valuable therapeutic agent for this group of disorders.

Irreversible eye diseases that cause blindness pose a major challenge today, as there is no fully effective cure; current therapies focus on slowing disease progression, managing symptoms, and preserving remaining vision. Our study aims to review research on the use of CoQ10 in eye diseases such as AMD, RP, and glaucoma, which can lead to irreversible vision loss and are associated with oxidative stress and mitochondrial dysfunction. This article examines whether CoQ10 can be a safe and effective adjunct in the treatment of the aforementioned conditions. Additionally, we provide directions for future research and explain how CoQ10 works in the studies discussed in this review. The PubMed and Google Scholar databases were searched using the keywords “coenzyme Q10”, “age-related macular degeneration”, “retinitis pigmentosa”, and “glaucoma”, focusing on research published from 2000 to 2025.

## 2. Characteristics of CoQ10

### 2.1. Chemical Structure

**Figure 1:**
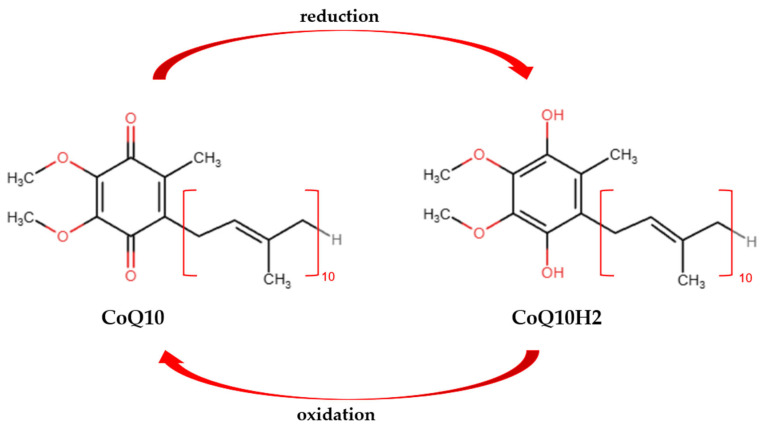
Chemical structures of CoQ10 and CoQ10H2.

CoQ10 is a fat-soluble antioxidant generated naturally within human cells. Its structure features a quinone core, specifically a benzoquinone ring, with a side chain composed of 10 polyisoprenoid subunits, which is typical for humans [16]. The benzoquinone ring in CoQ10 is derived from tyrosine, whereas the side chain originates from acetyl-CoA through the mevalonate pathway, a process also involved in cholesterol and lipid synthesis [20]. In various animal species, the number of polyisoprenoid subunits varies from 6 to 10 [16]. This molecule exists in two forms: an oxidized form (ubiquinone, CoQ10) and a reduced form (ubiquinol, CoQ10H2), as shown in Figure 1. For CoQ10 to participate in intracellular processes, ongoing conversion between its different redox forms is essential [21]. CoQ10H2 is a vital antioxidant that prevents the peroxidation of low-density lipoproteins in the blood. It also exhibits anti-inflammatory properties [22].

### 2.2. Molecular Mechanisms

CoQ10 is present in all cells, but its levels vary with the metabolic activity and energy needs of specific tissues and organs. The highest amounts of CoQ10 are found in heart muscle tissue, which explains its common use in cardiovascular disease supplementation [23]. Nevertheless, its anti-inflammatory, ROS-neutralizing, lipid membrane-stabilizing, and mitochondrial energy production-influencing effects may be significant in eye diseases. This section provides examples of the molecular mechanisms by which CoQ10 may operate.

CoQ10 can modulate key inflammatory pathways, translating into its broad therapeutic potential in diseases involving chronic inflammation. Although not all molecular mechanisms by which CoQ10 acts are well understood, it appears that this molecule may limit the production of pro-inflammatory cytokines by suppressing the expression of the gene that encodes nuclear factor kappa B (NF-κB) [24]. Furthermore, lipoperoxides in pathogens and oxidants generated during infection trigger a signaling pathway in monocytes that activates NF-κB [24]. This factor is responsible for activating genes for pro-inflammatory cytokines such as tumor necrosis factor α (TNF-α), interleukin-1 (IL-1), and interleukin-6 (IL-6) [25]. A meta-analysis of randomized clinical trials by Hou et al. [26] showed that CoQ10 supplementation can significantly lower the circulating levels of CRP, IL-6, and TNF-α in the general population. Furthermore, the researchers suggested that the optimal dose for this effect is 300–400 mg of CoQ10 per day [26].

ROS-induced oxidative damage is well known to alter the structure and function of proteins, carbohydrates, RNA, and DNA, as well as damage cell and mitochondrial membranes. Excessive ROS levels impact several signaling pathways, including nuclear factor erythroid 2 (Nrf2) and peroxisome proliferator-activated receptor (PPAR). These pathways form a positive feedback loop that regulates the expression of antioxidant, pro-survival, anti-inflammatory, and anti-apoptotic proteins [27]. Studies on rats with methotrexate (MTX)-induced testicular damage showed that adding CoQ10 to this treatment lowered the levels of malondialdehyde (MDA), myeloperoxidase, TNF-α, IL-6, and IL-1β [27]. It also reduced the Bax: Bcl2 ratio and increased glutathione and catalase (CAT) levels compared with MTX alone. CoQ10 boosted the MTX-induced suppression of Nrf2 and PPAR-γ signaling. It also affected downstream targets such as inducible nitric oxide synthase (iNOS), NF-κB, Bax, and Bcl-2. CoQ10’s protective effect stems from its role as an intracellular antioxidant, which prevents ROS-induced oxidative damage by activating the Nrf2 pathway. The research findings underscore the critical role of redox-sensitive Nrf2 in protecting cells against oxidative stress. Nrf2 regulates the activity of various factors, including nicotinamide adenine dinucleotide phosphate (NADPH)-producing enzymes, S-glutathione transferase, heme oxygenase-1 (HO-1), superoxide dismutase (SOD), and the Nrf2-PPAR-γ loop. Research indicates that PPAR-γ and Nrf2 are interconnected through a positive feedback mechanism that sustains and modulates the expression of multiple antioxidant and pro-oxidant genes in response to oxidative challenges. Moreover, the ligand-induced downregulation of PPAR-γ enhances the expression of eNOS and iNOS. These enzymes generate nitric oxide (NO), which can react with O_2_^−^ to produce highly reactive peroxynitrite, thereby suggesting a protective function of PPAR-γ against it [27]. This study demonstrates that CoQ10 supports the Nrf2-PPAR-γ signaling loop and its associated pathways, which reduce oxidative stress-induced damage and enhance testicular function. It also highlights CoQ10’s potent anti-inflammatory effects [27].

Another anti-inflammatory mechanism of CoQ10 involves its apparent ability to increase adiponectin levels, which, in turn, reduces the inflammatory response associated with TNF-α [24]. Zhang et al. [28], in a randomized, double-blind, placebo-controlled trial with 101 subjects with dyslipidemia, showed that CoQ10 supplementation increases adiponectin, a protective adipokine with anti-inflammatory properties. These studies also demonstrated that CoQ10 improves the glucolipid profile by mediating adiponectin. Adiponectin gene expression is tightly regulated by several factors, including PPAR-γ, which is primarily expressed in adipose tissue. Studies suggest that CoQ10 increases PPAR-γ expression, the primary positive regulator of adiponectin gene expression. Conversely, inflammatory factors like TNF-α inhibit adiponectin gene expression; this process is a molecular target for CoQ10 [28].

The antioxidant capacity of CoQ10 is crucial for reducing free radical production, thereby decreasing MDA levels. In addition, an increase in the activity of antioxidant enzymes that neutralize free radicals, such as SOD, glutathione peroxidase (GPx), and CAT, has been observed after CoQ10 supplementation [29]. Although the mechanism is not thoroughly comprehended, it is posited that CoQ10 safeguards these enzymes through the absorption of free radicals and enhances their functionality. Additionally, CoQ10 elevates the expression of genes encoding antioxidant enzymes and, by mitigating oxidative stress, augments total antioxidant capacity (TAC) levels [29].

Because oxidative stress negatively impacts in vitro and in vivo reproductive procedures, Florou et al. [30] conducted a meta-analysis of randomized clinical trials evaluating the impact of CoQ10 supplementation on fertility outcomes in women undergoing assisted reproductive technology (ART) procedures. The researchers demonstrated that CoQ10 supplementation increases the number of clinical pregnancies compared to placebo and no treatment in women undergoing ART [30]. Due to its antioxidant properties, CoQ10 is also used to combat oxidative stress in male infertility [31].

CoQ10 is crucial for adenosine triphosphate (ATP) production, as it transfers electrons from complexes I and II to complex III in the mitochondrial respiratory chain [22]. Oxidative phosphorylation is vital in the system, comprising five enzyme complexes and two mobile electron carriers that function within the mitochondrial respiratory chain [32]. This process couples the oxidation of reducing equivalents in mitochondria with proton gradient formation and dissipation across the inner mitochondrial membrane, fueling ATP synthesis. As a fundamental energy carrier in almost all cellular activities, ATP’s proper function is essential, and disruptions in this process are linked to numerous diseases [32]. CoQ10 neutralizes the ROS generated during electron transport, thereby protecting mitochondrial proteins, lipids, and DNA from oxidative damage [33]. Nevertheless, the mechanisms underlying CoQ10’s effect on mitochondrial function are more complex.

Stone et al. [34] observed increased peroxisome proliferator-activated receptor-γ coactivator 1-α (PGC1α) activity in the cell nucleus after 4 weeks of CoQ10 supplementation in pigs with hibernating myocardium (HM). In addition, PGC1α increases the expression of electron transport chain proteins in the mitochondria of HM tissue [34]. Since the transcription activator PGC1α is a crucial component of energy metabolism homeostasis, it is strongly associated with mitochondrial function. Additionally, PGC-1α reacts to environmental and intracellular stimuli and is regulated by sirtuin 1/3 (SIRT1/3), mitochondrial transcription factor A (TFAM), and adenosine monophosphate-activated protein kinase (AMPK), all of which are key regulators of mitochondrial biogenesis and performance [35].

Another interesting signaling pathway that CoQ10 may influence is AMPK–Yes-associated protein (YAP)–Optic atrophy 1 (OPA1) [36]. To test whether CoQ10 is involved in this pathway for treating atherosclerosis, the researchers used gene-silencing technology. After silencing AMPK, YAP phosphorylation was blocked, OPA1 expression decreased, and total ATP content dropped. Conversely, ROS levels and inflammatory factor expression increased overall. More importantly, after AMPK silencing, the therapeutic effect of CoQ10 was significantly reduced compared to before. Then, the YAP gene was silenced, and the related indicators were examined. Similarly, after YAP silencing, CoQ10’s therapeutic effect was significantly reduced [36]. This study demonstrates that CoQ10 enhances mitochondrial function and energy metabolism via the AMPK-YAP-OPA1 pathway while also reducing ROS.

Other studies have shown that CoQ10 and calcium supplementation effectively prevented muscle spasms resulting from the long-term toxicity of Hedgehog (Hh) pathway inhibitors, such as vismodegib or sonidegib, used in the treatment of basal cell carcinoma (BCC) [37]. Hh plays a key role in embryonic development stages, including cell development, differentiation, and organogenesis. In addition, this pathway promotes mitochondrial stress via the miR-625-5p/transient receptor potential melastatin-2 (TRPM2) axis, which promotes prostate cancer development in in vivo studies [38]. It therefore appears that CoQ10 may potentially influence this cellular pathway.

Wang et al. [39] showed that NAD(P)H quinone oxidoreductase 1 (NQO1) enhances bone formation and suppresses blood vessel formation in dental pulp stem cells (DPSCs) by modulating the mitogen-activated protein kinase (MAPK) pathway. They also found that CoQ10 can reverse the effects of NQO1 knockdown on these processes. Moreover, NQO1 decreases MAPK signaling, but this effect is offset by CoQ10 supplementation in DPSC-NQO1sh [39]. In another study, CoQ10 inhibited receptor activator of nuclear factor κB ligand (RANKL)-induced osteoclastogenesis by enhancing autophagy via the inactivation of the phosphoinositide 3-kinase/protein kinase B/mammalian target of rapamycin (PI3K/AKT/mTOR) and MAPK pathways in RAW264.7 cells [40]. This discovery could be of considerable importance for the therapeutic approach to postmenopausal osteoporosis.

**Figure 2:**
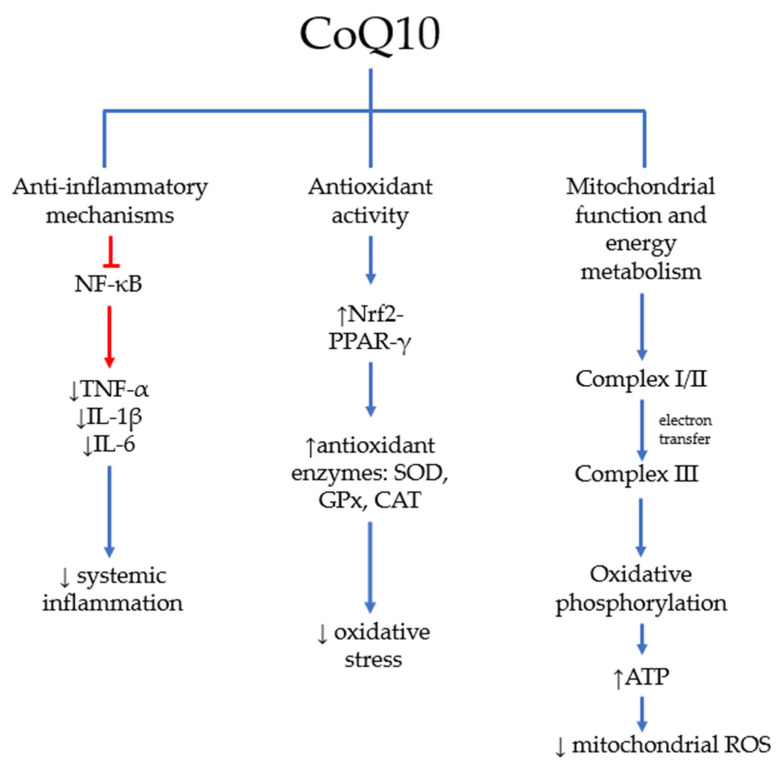
The main mechanisms of CoQ10 in the human organism.

As illustrated above, CoQ10’s activity in cells and tissues is highly complex, engaging numerous signaling pathways in its therapeutic effects. These examples highlight the significant benefits that CoQ10 supplementation can offer in various chronic diseases. Figure 2 illustrates the main mechanisms by which CoQ10 functions in the human organism.

### 2.3. Safety and Toxicity

Numerous clinical trials on CoQ10 have assessed its effectiveness, potential therapeutic applications, and its safety and toxicity. The exact daily requirement for CoQ10 is generally estimated at around 500 mg [41]. This estimate relies on a total body CoQ10 pool of roughly 2000 mg and an average tissue turnover period of four days. Furthermore, approximately 5 mg of CoQ10 is ingested daily through diet, with the majority of the body’s CoQ10 being produced internally [41]. Unfortunately, this synthesis can be disrupted due to primary and secondary CoQ10 deficiencies and a decline in production efficiency with age [41].

The range of CoQ10 doses in studies varies greatly. In their meta-analysis of randomized clinical trials, Xu et al. [42] assessed the safety of CoQ10 in patients with heart failure. These patients were administered CoQ10 in doses between 30 mg and 400 mg daily. The minimum and optimal doses of CoQ10 were not determined. Nevertheless, the safety data did not show an increase in adverse events, confirming the good tolerance of CoQ10 as an adjunctive therapy [42]. The literature indicates that CoQ10 is typically administered in doses of 200 to 300 mg/day for 3 to 6 months [41]. Some studies, however, have employed significantly higher doses (up to 2700 mg/day) or extended treatment durations (up to 5 years). Despite these differences, no serious side effects from CoQ10 supplementation were reported [41]. No data on the toxicity of CoQ10 were found either.

When evaluating the safety and toxicity of CoQ10 in ophthalmology, different forms should be considered, such as oral preparations and eye drops. An example demonstrating CoQ10’s high safety profile is its application in eye drops for retinopathy of prematurity (ROP). In these studies, CoQ10 was administered topically to infants born before 37 weeks of gestation [43]. Oral supplementation with CoQ10 in olive oil in rat pups exposed to retinal oxidative stress confirmed the safety and efficacy of this preparation in an animal model. However, as the researchers rightly pointed out, the use of oral forms of CoQ10 in premature infants requires further safety studies and the development of an optimal dose [44]. Tredici et al. [45] analyzed the safety and effectiveness of eye drops containing cross-linked hyaluronic acid, CoQ10, and vitamin E d-α-tocopheryl polyethylene glycol succinate (TPGS) in swimmers. The goal was to reduce eye irritation and repair damage from prolonged exposure to chlorinated water. Their study found no adverse effects from the long-term use of this solution [45]. Eye drops containing CoQ10 as an adjuvant therapy to standard treatments for neurotrophic corneal ulcers, postinfectious corneal ulcers, and Stevens–Johnson syndrome did not exhibit any adverse events during the observation period [46]. Interestingly, Titley et al. [47] studied the effects of different CoQ10 concentrations on immortalized human corneal endothelial cells (HCEC-12). They found that higher concentrations (0.5% and 1%) increased cell death, while lower concentrations (0.1% and 0.2%) were optimal for ex vivo studies. The potential cytotoxicity of high CoQ10 levels in clinical use could lead to serious side effects if toxic doses are administered or if CoQ10 builds up in tissues after initial safe doses [47]. Despite these findings, the researchers emphasized the need for further research in this area, highlighting the potential benefits of CoQ10 in corneal endothelial disease. Ophthalmological studies have not clearly established a safe and effective dose or concentration of CoQ10.

## 3. Pathogenesis of Selected Irreversible Eye Conditions

### 3.1. AMD

AMD is the leading cause of irreversible vision loss among people over 60 in developed countries. AMD is a chronic degenerative disorder affecting the central part of the retina—the fovea—resulting in progressive and painless central vision loss, with preserved peripheral vision. It is estimated that by 2040, the worldwide prevalence of any stage of AMD will rise from 196 million in 2020 to 288 million [48]. AMD is characterized by the progressive degeneration of photoreceptors and the retinal pigment epithelium, as well as the accumulation of retinal deposits called drusen. The disease typically begins in the non-neovascular (non-NV) form in the vast majority of patients and may progress to the neovascular (NV) type. The NV or wet form develops in approximately 10–15% of AMD patients and is responsible for nearly 90% of severe vision impairment cases due to the disease [49]. The main risk factor for AMD is older age, with others including genetic predisposition (e.g., CFH and ARMS2/HTRA1 polymorphisms), smoking, white race, hypertension, elevated total serum cholesterol, obesity, cardiovascular disease, micronutrient deficiency, and exposure to visible light [50,51,52].

AMD is characterized by the dysfunction and progressive degeneration of the central macular region involving the photoreceptor cells in the outer retina, the retinal pigment epithelium (RPE), Bruch’s membrane, and the choroid. In the early stages of the disease, extracellular debris composed of glycoproteins and lipids accumulates in Bruch’s membrane, forming structures called drusen. The pathogenetic mechanisms underlying AMD progression involve mitochondrial dysfunction, oxidative stress, alterations in proteolysis and lipid homeostasis, disturbances in extracellular matrix (ECM) metalloproteinase systems, and chronic inflammatory response with complement system activation. Mitochondrial dysfunction in the RPE results in decreased ATP production and oxidative stress through excessive ROS generation [13,53]. Oxidative stress in RPE cells leads to defects in autophagy and the proteolysis of waste products generated during the renewal of photoreceptor outer segments. Insufficient protein clearance contributes to the accumulation of waste lipidic and protein aggregates [54]. The aggregation of misfolded molecules in drusen can activate inflammatory responses and promote angiogenesis in the subretinal region [55]. Inflammation in AMD is mediated by innate immunity pathways, including complement system activation with the formation of membrane attack complex (MAC), microglial and vascular endothelial activation, increased cytokine production, alterations in the ECM metalloproteinase system, and the infiltration of macrophages that are an important source of proangiogenic and profibrotic markers [56,57]. Importantly, innate immunity pathways participate in aggregation and lipid homeostasis. They also respond to oxidative stress with mitochondrial dysfunction and involve changes in the ECM metalloproteinase system. Additionally, they contribute to inflammatory responses associated with chronic inflammation, significantly involving the complement system. The chronic inflammatory process in drusen drives the progression of AMD to its advanced stages through the activation of vascular endothelial growth factor (VEGF) and LTB4, especially in individuals with genetic and local susceptibility [53]. Consequently, non-NV AMD converts to NV AMD, which is associated with neovascularization and RPE loss.

### 3.2. RP

RP is an inherited retinal dystrophy that causes the progressive loss of photoreceptor function and survival, initially affecting rod cells and later cone cells. RP is the most common genetic retinal disease, affecting over 1.5 million individuals worldwide [58]. Typical clinical presentation includes night blindness (nyctalopia) and progressive peripheral vision loss (tunnel vision), which can result in complete blindness by the age of 40 [59]. The precise mechanism of photoreceptor destruction in RP is multifactorial and closely linked to the causative mutation underlying the development of the disease. More than 200 genes associated with RP have been identified to date, and the pattern of inheritance can be autosomal dominant, autosomal recessive, or X-linked. The mechanisms of photoreceptor death in RP include intrinsically or extrinsically induced apoptosis, regulated necrosis, and autophagy. Among the most important mechanisms underlying rod photoreceptor damage in RP are oxidative stress, high levels of cyclic guanosine-3′,5′-monophosphate (cGMP), calcium ion overload, endoplasmic reticulum (ER) stress, and inflammatory responses [60,61]. Photoreceptors are characterized by high metabolism, abundant mitochondria, and high oxygen demand. The increased production of ROS with a decreased antioxidant defense system modifies nucleic acids, proteins, and lipids, promoting rod and cone degeneration in RP. In response to oxidative stress, activated microglia release pro-inflammatory cytokines and chemokines, additionally increasing extracellular ROS in the outer nuclear layer, and promote the synthesis of complement components [62,63]. Furthermore, activated microglia may induce cone death and the degeneration of rods in the later stages of RP. Mutations causing impaired PED6 function lead to elevated cGMP levels, which result in uncontrolled calcium influx into cells via cyclic nucleotide-gated channels, causing the ER stress-induced apoptosis of photoreceptors [64]. Intracellular calcium accumulation leads to the apoptosis of photoreceptors by caspase-dependent mechanisms or caspase-independent cell death due to the activation of proteolytic calpain enzymes, triggering lysosomal membrane permeabilization.

### 3.3. Glaucoma

Glaucoma impacts more than 80 million individuals globally and is a major cause of permanent vision loss. The main risk factors for glaucoma include age, genetic predisposition, smoking, race, male sex, and elevated intraocular pressure (IOP) [65]. Glaucomatous degeneration initially affects retinal ganglion cells (RGCs) and their axons, which form the optic nerve. RGC damage in glaucoma with elevated IOP results from reduced ocular blood flow, the activation and hypertrophy of retinal astrocytes, the overexpression of aquaporin 4, and mutant superoxide dismutase. Astrocytes activated by altered inflammatory signals are unable to perform their vital functions. It has been shown that abnormal astrocyte activity may also contribute to RGC apoptosis through a glutamate excitotoxic mechanism in normal tension glaucoma [66,67]. Furthermore, increased IOP glaucoma reduces retinal metabolic activity and leads to mitochondrial dysfunction with elevated ROS production. This results in chronic oxidative stress, which initiates altered inflammatory responses, contributing to astrocyte activation and ECM remodeling [68]. Elevated IOP activates transient receptor potential channel vanilloid 4 (TRPV4), which is intensively expressed in bipolar cells and RGC. Activated TRPV4 mediates Ca^2+^ and Na^+^ influx, leading to glutamate release by bipolar cells and to excitotoxicity in RGC [69].

## 4. CoQ10 in Irreversible Eye Conditions

Given the increasing prevalence of eye disorders in aging populations, the search for effective, accessible, and safe treatment options is of growing importance. In this context, CoQ10 has emerged as a promising candidate, owing to its well-established antioxidative properties and favorable safety profile [70]. The potential application of CoQ10 in eye diseases has been investigated in both in vivo and in vitro studies, as well as in clinical trials. This section presents the latest research findings in this field.

Over 20 years ago, researchers demonstrated that free radicals significantly contribute to the development of AMD. This was seen during the examination of CoQ10 levels in the plasma and blood platelets of patients with exudative AMD and a matched control group [71]. They also observed that CoQ10 could offer protective effects against the disease, which sparked greater interest in this natural antioxidant within ophthalmology. Another promising randomized, double-blind, placebo-controlled clinical trial by Feher et al. [72] was conducted to evaluate the efficacy of a combination of acetyl-L-carnitine (ALC), n-3 fatty acids (n-3 FAs), and 10 mg of CoQ10 on visual function and fundus changes in early-stage AMD. A total of 106 patients with a clinical diagnosis of early AMD were divided into a study group and a control group. They were monitored over 12 months for changes in mean visual field defect (VFMD) from baseline, along with secondary efficacy measures such as visual acuity (VA; using the Snellen and ETDRS charts), foveal sensitivity measured by perimeter, and fundus changes evaluated based on the International AMD Classification and Assessment System criteria. At the end of the study, the study group showed significant improvements in all four visual function parameters, with only 1 in 48 cases (2%) showing deterioration in VFMD. Importantly, in the control group, a deterioration in this parameter was observed in 17% of the subjects. More promising results compared to placebo were seen in the analysis of the reduction in the area of the treated eyes covered with drusen [72]. The researchers highlighted that the combination of compounds affecting mitochondrial lipid metabolism can enhance and stabilize visual function. Additionally, it may also improve changes in the fundus in patients with early AMD. Analyzing the results above makes it hard to pinpoint CoQ10’s exact role in the combination. However, it seems that these ingredients work together synergistically and share a common mechanism of action.

Oxidative stress and mitochondrial dysfunction within the RPE are well-known factors in many retinal disorders, such as AMD, DR, and RP. Hernandez et al. [73] investigated the influence of adding CoQ10 (Q) at a wide range of doses (0.01–100 µM) to a nutritional antioxidant complex—Nutrof Total (N)—on the H_2_O_2_-induced oxidative stress model in adult human RPE cells. Compared with the control, H_2_O_2_ significantly increased the levels of the marker of oxidative DNA damage, 8-hydroxy-2′-deoxyguanosine (8-OHdG); caspase-3, a marker of early apoptosis; and TUNEL, a marker of late apoptosis. In addition, H_2_O_2_ increased the levels of inflammatory mediators, including RANTES and caspase-1. Moreover, H_2_O_2_ increased the levels of superoxide, an estimate of ROS production, and dynamin-related protein 1 (DRP-1), known to be involved in mitochondrial fusion/fission and mitochondrial energy regulation. In contrast, H_2_O_2_ reduced the expression of genes such as *IL1β*, *SOD2*, and *CAT*, which are involved in inflammation and the oxidative stress response. Interestingly, Q demonstrated a remarkable recovery in *IL1β* gene expression, TUNEL, TNF-α, caspase-1, and JC-1 compared to H_2_O_2_, while NQ showed a significant synergistic effect in caspase-3, TUNEL, mtDNA, and DRP-1 [73]. The results demonstrate that treatment with CoQ10, a nutritional complex, or their combination mitigates oxidative stress-induced damage in cultured human RPE cells. The protective mechanism of the combined supplementation appears to involve antioxidant effects, the inhibition of apoptosis, and the maintenance of mitochondrial integrity. It may therefore be promising for the treatment of such common diseases as AMD. Nevertheless, further research is needed, particularly regarding bioavailability, distribution, and interactions among antioxidant molecules.

Ocular hypertension (OHT) is the primary risk factor for glaucoma. High IOP induces the chronic activation of retinal and optic nerve glial cells, which causes a pro-inflammatory state and disrupts the blood–retinal barrier, leading to the death of RGCs [74]. Matamoros et al. [75] in their study used a laser-induced mouse model of OHT. Rodents received daily oral CoQ10 (200 mg/kg) and citicoline (500 mg/kg) starting 15 days before laser treatment and continuing until sacrifice (3 days for retina, 7 days for visual pathway). The retina, dorsolateral geniculate nucleus, superior colliculus, and visual cortex were histologically examined. OHT and OHT-CitiQ10 eyes had increased IOP versus controls. CoQ10 and citicoline lowered IOP significantly within 24 h and at 3 days post-laser. Both OHT and treated groups showed increased macro- and microglia in retinal layers, but treatment reduced this increase, indicating decreased glial activation and pro-inflammatory cytokines, supported by lower expression levels. Glial activation in visual pathway tissues was also reduced [75].

In sum, the aforementioned compounds exhibit neuroprotective effects. A limitation is seen in the uncertainty about which of the administered components is responsible for the neuroprotective and anti-inflammatory effects. It appears that CoQ10 and citicoline act synergistically, as tested in the same OHT model by Matamoros et al. [76,77].

The researchers investigated the neuroprotective effects of CoQ10 and citicoline on RGCs and visual pathway neurons in a mouse model of unilateral, laser-induced OHT [76]. Mice were divided into four groups: vehicle (*n* = 6) with no procedures; citicoline + CoQ10 (*n* = 6); laser–vehicle (*n* = 6), which received neutral gelatine and had OHT; and laser–citicoline + CoQ10 (*n* = 6), which received 0.5 mL gelatine daily containing 500 mg/kg citicoline and 200 mg/kg CoQ10. Substances were administered 15 days before and 7 days after laser exposure, and then the mice were sacrificed. This study found that IOP increased after 48 h and 3 days in all groups except OHT–citicoline and CoQ10. At 5 days, OHT eyes showed higher IOP than the contralateral and vehicle groups, but not compared to OHT–citicoline + CoQ10. No differences appeared at 7 days. OHT eyes had fewer RGCs, and citicoline + CoQ10 reduced this loss. IOP negatively correlated with RGC loss in untreated mice but not in treated ones. The authors concluded that oral citicoline and CoQ10 protect RGCs in OHT mice [76].

Another recent study by this research group [77] confirmed that combining CoQ10 with citicoline has neuroprotective and anti-inflammatory effects in OHT. Sixty male mice were divided into four groups: vehicle (*n* = 12), CitiQ10 (*n* = 12), OHT (*n* = 18), and OHT + CitiQ10 (*n* = 18). In the treated groups, mice received 500 mg/kg of citicoline and 200 mg/kg of CoQ10. For both the OHT and OHT + CitiQ10 groups, the study was conducted on the left-lasered eye and the right eye without OHT induction. Compared with the rest of the eyes, eyes treated with citicoline and CoQ10 showed a decrease in IOP at 24 h and 3 days after OHT induction, but by 7 days post-induction, IOP in treated eyes was comparable with that in controls and contralateral eyes, suggesting that CoQ10 and citicoline supplementation have hypotensive effects in the acute phase. Regarding statistically significant differences in Retinal Nerve Fiber Layer (RNFL) thickness, in group OHT-CitiQ10 compared with its controls, there was an increase in RNFL thickness in the superior, inferior, and nasal sectors. The next examined parameter is particularly interesting because scientists analyzed the number of vitreous particles in the basal vitreous. At both 3 and 7 days after induction, OHT eyes showed a significantly higher number of vitreous particles than the vehicle group. Moreover, at 3 days post-induction—when particle levels peaked—a significant difference was observed between OHT eyes and their contralateral eyes [77]. In addition, when OHT eyes were compared with those receiving CitiQ10 treatment, the CitiQ10 group exhibited a markedly reduced number of vitreous particles at 3 days post-induction. In the untreated groups, IOP showed a strong positive correlation with the number of vitreous particles. In contrast, no such correlation was observed in the eyes treated with CitiQ10. The authors propose that the presence of vitreous particles may be associated with inflammation, and their reduction after the administration of these substances may reflect anti-inflammatory properties. The authors also used full-field electroretinograms (ffERGs) to assess functional alterations. In OHT eyes, at 3 days post-induction, they observed early functional impairment, particularly reduced scotopic b-wave and oscillatory potentials. In the CitiQ10-treated groups, ERG recordings showed only non-significant trends at the investigated time-points [77]. These results indicate that treatment with CitiQ10 may help reduce early retinal injury in glaucoma. Additionally, Optical Coherence Tomography (OCT) and ffERG appear to be effective methods for monitoring disease progression, highlighting the therapeutic promise of this strategy in the early stages of the condition. One limitation of the current study is that only the combined formulation of citicoline and CoQ10 was evaluated.

An interesting study evaluating retinal and cortical responses to visual stimuli was conducted by Parisi et al. [78] in 43 patients with open-angle glaucoma (OAG). By analyzing pattern electroretinogram (PERG) and Visual Evoked Potential (VEP) recordings at baseline, 6 months, and 12 months, their study compared the effectiveness of CoQ10 with vitamin E (two drops daily) as an add-on to β-blocker treatment in 22 patients (GC group) versus β-blocker-only therapy in 21 patients (GP group). At 6 and 12 months, the PERG and VEP response parameters in the GP group remained consistent with baseline measurements. Notably, in the GC group, the latencies of PERG P50 and VEP P100 were reduced, while the amplitudes of PERG P50-N95 and VEP N75-P100 increased significantly compared to baseline. Moreover, the changes in latencies and amplitudes in the GC group were considerably more pronounced than those in the GP group [78]. This study showed that CoQ10, when combined with vitamin E in OAG, has a beneficial effect on inner retinal function, resulting in improved responses in the visual cortex. The findings of this study are especially significant because they highlight the high clinical importance of antioxidants when used alongside standard therapy.

Another promising conclusion was drawn from a prospective, randomized clinical study by Dogan et al. [79]. The authors divided patients aged ≥18 years with primary open-angle glaucoma (POAG) into a study group (48 eyes of 48 patients) and a control group (48 contralateral eyes of the same patients). Both groups used eye drops containing timolol and dorzolamide. The study group also involved the topical application of CoQ10 and vitamin E TPGS twice daily for 12 months. The mean IOP in the study group was 16 mmHg, and in the control group, it was 16.5 mmHg. The inclusion criteria were: no history of cataract/glaucoma surgery, no history of neuritis or optic nerve damage, refraction from −3.0 D to +3.0 D, and astigmatism less than 2.5 D. After 6 and 12 months of administration, the analyzed exams and parameters were: VEP, Visual Field (VF), and OCT to evaluate the RNFL and Ganglion Cell Layer (GCL) thickness. After 12 months of treatment, GCL thickness decreased in both groups; however, the decrease was statistically significant only in the control group. This allows the conclusion to be drawn that the tested eye drops have a protective influence on the GCL. For the RNFL, the initial thickness in the study group was 79.9 μm, and in the control group, it was 79.7 μm. After 6 months of treatment, the thickness was 79.8 μm in the study group and 78.7 μm in the control group. At the endpoint, after 12 months of treatment in the study group, RNFL thickness was 79.4 μm; in the control group, it was 76 μm [79]. It is clear that the untreated group experienced a greater loss of RNFL. An examination of the next parameter, VEP, showed that after 12 months of supplementation, 24 eyes in the study group exhibited a significant reduction in P100 implicit time, along with an increase in amplitude. Among the control group, 26 patients exhibited a significant prolongation of P100 implicit time and a corresponding decrease in P100 amplitude. In conclusion, VF changes are important. In a study group after 12 months of therapy, the current VF was preserved, whereas in the control group, progression was observed [79]. As is well known, a patient’s ability to function primarily depends on VA and the maintenance of the VF. Therefore, the findings of this study need additional refinement. This study evaluates a wide range of tests we currently use to monitor glaucoma and demonstrates that CoQ10 and vitamin E may be effective as adjuvants in glaucoma therapy. Both substances are known for their potent antioxidant properties, and when used together as drops, they likely enhance each other’s anti-inflammatory effects.

An issue that needs attention due to its common occurrence is PEX syndrome (pseudoexfoliative syndrome) and its progression to glaucoma. In this type of elastosis, ECM material accumulates in the anterior part of the eye, particularly in the trabecular meshwork, thereby obstructing the outflow of aqueous humor and leading to increased IOP and PEX glaucoma. In a prospective, randomized clinical study, the authors investigated 64 eyes from 64 patients [80]. All patients underwent phacoemulsification and intraocular lens implantation surgery, during which aqueous humor was aspirated from the anterior chamber. They divided the patients into three groups: The first group consisted of patients with PEX glaucoma who received 100 mg CoQ10 and 500 mg vitamin E TPGS topically twice daily for 1 month preoperatively. The second group included patients with PEX glaucoma who did not receive the above preparation, and the third group consisted of patients with PEX syndrome without glaucoma. The key point was to evaluate the levels of oxidative stress markers, SOD and MDA, in these groups. It was found that in patients with PEX syndrome, the level of SOD was lower compared to the group with PEX glaucoma, including both treated and untreated patients. This seems reasonable, considering that glaucoma also involves an oxidative stress component. Therefore, it was not surprising that the supply of CoQ10 and E TPGS in the PEX glaucoma group led to a decrease in SOD levels, which were significantly lower compared to untreated PEX patients with glaucoma. The MDA levels did not show significant changes across the groups [80]. In this interesting case of PEX glaucoma, CoQ10 combined with other ingredients appears to be effective in reducing oxidative stress and warrants further research. 

In one of the studies, the researchers examined the effect of oral CoQ10 administration on a mouse model of glaucoma [81]. The mice were divided into four groups: D2-Gpnmb^+^ mice fed a control diet (*n* = 25), D2-Gpnmb^+^ mice fed 1% CoQ10 (1600–2000 mg/kg) (*n* = 25), DBA-2J mice fed a control diet (*n* = 70), and DBA-2J mice fed CoQ10 (*n* = 80). The treatment lasted for six months. The authors concluded that CoQ10 supplementation did not significantly affect IOP but improved other parameters. For instance, in the control glaucomatous groups, RGC loss was substantially higher, while the number of surviving RGCs was greater in the treated glaucomatous DBA/2J mice. Additionally, mice on a control diet experienced more optic nerve axon loss compared to the treated glaucomatous DBA/2J group, where neuroprotection was observed. An interesting finding is that untreated groups showed higher astroglial levels compared to treated groups, indicating that CoQ10 supplementation may prevent astroglial activation and optic nerve damage. In treated groups, CoQ10 inhibits NR1, NR2A, SOD2, and HO1 protein upregulation in glaucomatous DBA/2J mouse retinas, suggesting that glutamate excitotoxicity triggers oxidative stress, which may increase retinal vulnerability through NR involvement in neurodegeneration. Furthermore, CoQ10 may enhance RGC survival by affecting the mitochondria-related apoptotic pathway. Lastly, emphasizing the neuroprotective effect of CoQ10, it was found that in the retina of glaucomatous DBA/2J mice, CoQ10 administration decreased Bax levels and increased pBad protein expression, which are critical in apoptosis [81].

In RP, it is particularly noteworthy that, in some cases, non-syndromic RP is linked to a mutation in *COQ8B*, the gene involved in the biosynthesis of CoQ10 [14]. The researchers identified five individuals from four families with biallelic DNA changes in *COQ8B*. They suggest that the *COQ8B* product plays an essential role by exhibiting ATPase activity in the presence of CoQ10 intermediates. Likely, the depletion or inhibition of *COQ8B* results in decreased ubiquinone levels, leading to mitochondrial respiratory chain disorders, oxidative stress, and, in this case, the death of cones and rods, which manifests as RP. The above supports the hypothesis that CoQ10 plays a key role in maintaining retinal function and that its deficiency and supplementation deserve attention in developing treatments for diseases such as RP. Pharmacokinetic issues were also discussed—due to the poor penetration of the coenzyme through natural barriers—prompting further research on the intravitreal administration of ubiquinone, similar to the approaches used in AMD and with anti-VEGF injections [14].

Research on a porcine retinal degeneration model showed that CoQ10 provides neuroprotection against oxidative stress-induced retinopathies [82]. In this study, a porcine organ culture was divided into three groups over eight days: control, H_2_O_2_-induced oxidative damage, and H_2_O_2_-induced oxidative damage with CoQ10 treatment. On day one, the latter two groups’ tissues were exposed to 500 µM of H_2_O_2_ for 3 h. The last group received CoQ10 therapy—700 µM diluted in Lutrol—for 48 h. An immunohistological analysis of RGCs and microglia served as neurodegeneration markers. Retinal thickness was similar across all groups, but microglial activation was observed only in the H_2_O_2_ group, which also showed significant RGC loss. Conversely, the CoQ10-treated group exhibited a protective effect on RGCs. Oxidative stress was assessed via gene expression involved in intracellular protection. *NRF2* mRNA levels rose significantly after oxidative stress, with higher levels in the CoQ10 group than in controls. The H_2_O_2_ + CoQ10 group did not show a significant increase in *NRF2* compared to the H_2_O_2_ group. *SOD2* levels, another oxidative stress marker, were markedly elevated only in the H_2_O_2_ group, but CoQ10 treatment normalized these levels [82]. The authors suggested that CoQ10 protects RGCs by inhibiting apoptosis-related pathways, activating intracellular protective mechanisms, and reducing mitochondrial stress.

Focusing on oxidative stress, ischemic retinopathies are among the retinal pathologies with still inadequate treatment options, and they can have severe consequences. A retrospective clinical case study reported [83] on 48 patients receiving vitamins and CoQ10 supplementation, including those with non-arteritic anterior ischemic optic neuropathy (NAION) (*n* = 18), retinal artery occlusion (RAO) (*n* = 7), and visual deficits like hemianopia or quadrantanopia caused by stroke (*n* = 10), as well as other conditions such as optic nerve atrophy, cone dystrophy, RP, and various retinal vascular occlusions. Specific details about these injuries are not provided. These patients began vitamins and antioxidant therapy, including CoQ10 at a dose of 100 mg/day, after diagnosis. The doses, composition, and duration varied. Diagnosis involved multiple assessments: Visual Field Index (VFI), OCT of RNFL and macula, VA, evoked potentials, fluorescein angiography, examination of the optic disk and anterior segment, indirect ophthalmoscopy, and neurological studies, including MRI when necessary. The VFI was chosen as the primary evaluation metric, which is justified since patients typically evaluate treatment success through VA and VF [83]. The authors found that most patients showed improvement in the VF, with the difference being statistically significant. Among all studied patients, those with RAO experienced the greatest increase in the VFI progression rate. Similar conclusions were drawn for patients with NAION. Conversely, patients with homonymous hemianopia or quadrantanopia after stroke exhibited significantly higher VFI progression rates in both eyes following treatment [83]. Based on the data from this study, it is difficult to determine the specific effect of CoQ10 alone on the examined vision parameters, as multi-component preparations were tested at various doses and for different treatment periods. Nevertheless, the improvement in the assessed parameters seems to support the recommendation of CoQ10 and vitamins supplementation in eye diseases. Table 1 summarizes the results of the studies discussed in this section.

**Figure 3:**
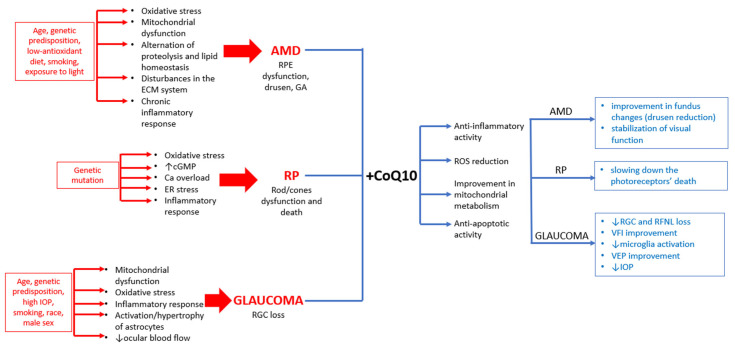
Risk factors of AMD, RP, and glaucoma and potential benefits of CoQ10 in their therapy.

Figure 3 summarizes risk factors of AMD, RP, and glaucoma, along with the potential benefits of CoQ10 supplementation in these conditions, based on the studies discussed in this section.

## 5. Limitations

Despite promising research, certain limitations must be considered when interpreting these findings. A major limitation of this review is the limited number of studies examining CoQ10 as a single agent for the specified ophthalmic conditions. There is a noticeable lack of large-scale clinical trials, and the studies differ in CoQ10 doses and trial durations. Importantly, none of the studies discussed above reported significant adverse effects, highlighting the safety of daily CoQ10 supplementation. Although many studies have confirmed CoQ10’s safety, questions about the optimal dosage and long-term effects remain and require further investigation, especially given the potential risk of CoQ10 buildup in tissues.

Another important aspect to consider is the pharmacokinetics of CoQ10. Because of its hydrophobic nature and high molecular weight, its absorption from the diet is slow and limited [84]. Formulations that solubilize CoQ10 in dietary supplements can enhance bioavailability. T(max) is around 6 h, with an elimination half-life of roughly 33 h. In healthy adults, plasma CoQ10 levels generally range from 0.40 to 1.91 µmol/L [84]. Some studies in this review used oral preparations, while others employed eye drops, which may also influence the bioavailability and effectiveness of the active ingredients. While assessing the pharmacokinetics of substances in oral formulations is relatively straightforward, studying the pharmacokinetics of substances in ophthalmic formulations can be quite challenging due to the eye’s unique barriers and the difficulty in evaluating how the substance interacts with ocular tissues. Researchers highlight the eye’s unique structure, with extensive compartmentalization and limited tissue permeability [85]. It contains barriers like the tear film, corneal epithelium, conjunctiva, sclera, and retina. The corneal epithelium mainly blocks hydrophilic drugs, allowing only small lipophilic molecules to pass. Blood–ocular barriers, including the blood–retinal and blood–aqueous barriers, restrict systemic drug entry. Active transporters, such as P-glycoprotein and enzymes in ocular tissues, further influence drug pharmacokinetics and contribute to interindividual variability [85]. Therefore, it seems important to seek new physiological pharmacokinetic models for these formulations as well as new drug delivery systems. Recent innovations of CoQ’s formulations include sustained-release eye drops and ion-activated gel microemulsions [86]. Advances over recent years have led to nanoparticle-based systems and polymeric micelles, which can greatly improve the absorption and efficacy of this antioxidant [87,88]. An additional promising oral formulation is orally disintegrating tablets (ODTs) with CoQ10 granules made by spray drying, hot-melting, and wet granulation, enhancing bioavailability and pharmacokinetics [89]. Overall, creating suitable formulations for CoQ10 supplements remains a key challenge for researchers.

Most studies focus on complex formulations, which complicates attributing therapeutic effects exclusively to CoQ10. It is highly probable that these ingredients work synergistically, emphasizing the need for research on formulations containing only CoQ10 specifically for ophthalmology. Often, the studies cited in this review used a combination of CoQ10 and vitamin E TPGS, underscoring the importance of potent antioxidants in eye drops. Using a combination of these substances in eye drops seems especially effective because it addresses CoQ10’s poor water solubility and limited ability to penetrate the cornea. TPGS functions as a solubilizer and inhibits P-glycoprotein efflux pumps in corneal cells, which enhances the absorption and delivery of CoQ10 to the retina [78]. Since many compounds in combined formulations are fat-soluble, and most oral preparations are oil-based capsules, absorption by the body is increased. Additionally, the combination of CoQ10 and citicoline proves highly effective, as studies suggest that they perform better together than separately. However, the existing literature lacks data regarding the efficacy of CoQ10 as a standalone treatment for ocular diseases. Most research focuses on multi-compound combinations that exhibit synergistic effects, thereby complicating the elucidation of their precise mechanisms under specific conditions.

## 6. Conclusions

The research findings support the important role of antioxidants like CoQ10 as a complementary therapy for irreversible eye conditions such as AMD, RP, and glaucoma. The studies highlight that combining CoQ10 with vitamin E TPGS in eye drops and with citicoline orally is especially effective. Clearly, these supplementary substances boost CoQ10’s therapeutic capabilities and increase its overall impact. Nevertheless, the noticeable lack of studies using CoQ10 as monotherapy makes it difficult to interpret the results and properly assess the efficacy of this antioxidant in the therapy of eye diseases.

In glaucoma research, in vivo mouse studies show that combining CoQ10 with citicoline reduces IOP, enhances RNFL thickness, and boosts RGC survival. Additionally, it decreases vitreous particles and astroglial activation. Clinical trials further reveal improvements in VEP, preserved VF, and lowered inflammation and oxidative stress markers.

In the case of AMD, there have been confirmed improvements in visual functions, a decrease in VFMD worsening, and a reduction in drusen, although there is a notable lack of studies focusing solely on CoQ10. A similar situation exists for RP. However, in vitro studies using a porcine model of retinal degeneration show promising results, with CoQ10 reducing microglia levels, decreasing RGC loss, lowering oxidative stress markers, and increasing protective factors against oxidative stress. Additionally, other studies combining CoQ10 with different antioxidants or vitamins have also demonstrated an improved VFI.

Based on the research findings, we believe that supplementing with CoQ10 for patients with AMD, RP, and glaucoma can offer numerous benefits, including enhanced vision parameters and overall health, both of which decline with age. Future researchers should address concerns about the safety and toxicity of CoQ10, as well as obstacles in developing suitable formulations, which are related to the pharmacokinetics of ophthalmic drugs. Researchers are also advised to conduct studies on CoQ10, both alone and combined with standard pharmacological treatments, to assess its effectiveness in treating eye diseases.

## Figures and Tables

**Table 1:** Summary of research results concerning CoQ10 in selected ophthalmological conditions.

Authors	Study Type	Group Characteristics	COQ10 Formulation Characteristics	Results
Feher et al. [72]	Clinical trial	106 patients with early-stage AMD	10 mg of CoQ10 with 100 mg ALC, 530 mg n-3 FA or soy oil; 2 capsules per day for 12 months	Improvements in visual functions; ↓ VFMD worsening; ↓ in a drusen-covered area.
Hernández et al. [73]	In vitro	H_2_O_2_-induced oxidative stress model in adult human RPE cells	CoQ10 (0.01–100 µM) + Nutrof Total	Q: recovery in *IL1β* gene expression, TUNEL, TNFα, caspase-1, and JC-1 vs. H_2_O_2_; NQ: synergist effect in caspase-3, TUNEL, mtDNA, and DRP-1.
Matamoros et al. [75]	In vivo	Murine model of laser-induced OHT	Orally, gelatine contained 500 mg/kg of citicoline and 200 mg/kg of CoQ10, once daily	↓ in IOP at 24 h and 3 days post-laser in the study group compared with the non-treated group; ↓ indications of micro- and macroglial activation in the retina and visual pathway tissues.
Matamoros et al. [76]	In vivo	Murine model of laser-induced OHT	Orally, gelatine contained 500 mg/kg of citicoline and 200 mg/kg of CoQ10, once daily	↓ IOP; ↓ RGC loss.
Matamoros et al. [77]	In vivo	Murine model of laser-induced OHT	Orally, gelatine contained 500 mg/kg of citicoline and 200 mg/kg of CoQ10, once daily	↑ RNFL thickness; ↓ number of vitreous particles; Mild ↓ IOP;
Parisi et al. [78]	Prospective trial	43 patients with OAG	0.1% CoQ10 and 0.5% vitamin E TPGS eye drops; 2 drops per day for 12 months	PERG and VEP improvement.
Dogan et al. [79]	Clinical trial	48 eyes of 96 patients with POAG	0.1% CoQ10 and 0.5% vitamin E TPGS eye drops, twice a day for 12 months	Less loss of GCL and RNFL in the treated group; Significant improvement in VEP parameters; Preserved VF in the treated group.
Ozates et al. [80]	Clinical trial	64 patients with PEX glaucoma or PEX syndrome without glaucoma	0.1% CoQ10 and 0.5% vitamin E TPGS eye drops; 2 drops daily for 1 month	↓ SOD levels in the PEX glaucoma group; No changes in MDA.
Lee et al. [81]	In vivo	Mouse model of glaucoma	1% CoQ10 diet for 6 months	↑ RGC survival; preserved optic nerve axons; ↓ astroglial activation; ↓ Bax levels; ↑ pBad protein expression; Inhibition of the upregulation of NR1/NR2A, SOD2, and HO1 protein expression in the retina.
Leoni Deppe et al. [82]	In vitro	H_2_O_2_-induced oxidative damage in porcine retinal degeneration model	700 µM of CoQ10 diluted in Lutrol	In the treated group: ↓ levels of microglia, ↓ RGC loss, ↓ oxidative stress markers, and ↑ protective factors against oxidative stress.
Fernández-Vega B et al. [83]	Retrospective case series	Ischemic retinopathy caused by RAO	CoQ10 100 mg/day orally, vitamins (unspecified)	Improved VFI; the mean improvement is +22 ± 17% per year.

↓ Decrease; ↑ Increase.

## Data Availability

No new data were created or analyzed in this study. Data sharing is not applicable to this article.
